# Improving behavioural interventions in healthcare to address the needs of disadvantaged groups: A policy-focused evidence brief

**DOI:** 10.1016/j.puhip.2026.100780

**Published:** 2026-03-26

**Authors:** Anna Gkiouleka, Sashika Harasgama, Helen Pearce, Lucy Johnson, Isla Kuhn, Amy Dehn Lunn, Danielle Lamb, Payam Torabi, Alice Vodden, Serge Engamba, Jack Birch, Adnaan Ghanchi, John Ford

**Affiliations:** a Wolfson Institute for Population Health, Queen Mary University of London, UK; b University of Cambridge Medical Library, School of Clinical Medicine, Cambridge, UK; c NIHR Applied Research Collaboration, UCL, UK; d The Aberfeldy Practice, UK; e University College London Hospitals NHS Foundation Trust, UK; f Exeter Collaboration for Academic Primary Care (APEx), University of Exeter, UK; g NIHR Policy Research Unit Behavioural and Social Sciences, Population Health Sciences Institute, Faculty of Medical Sciences, Newcastle University, Newcastle upon Tyne, UK; h Department of Public Health and Primary Care, University of Cambridge, UK

**Keywords:** Health inequalities, Behavioural interventions, Chronic disease prevention

## Abstract

**The policy challenge:**

Chronic conditions like cardiovascular disease and Type 2 Diabetes share common modifiable risk factors. Although behavioural interventions can help in reducing these risks in the overall population, they are often less effective for disadvantaged groups and may risk worsening inequalities. A systemic approach is needed, with healthcare systems playing a key role in adapting such interventions to be more inclusive and accessible to those who need them the most. This policy-focused evidence brief explores international evidence on improving behavioural interventions for disadvantaged groups.

**Key evidence to inform policy:**

There is no single set of specific interventions that can guarantee improved access, uptake and effectiveness of behavioural interventions among disadvantaged groups. However, effective interventions share four key features: they 1) adopt a targeted approach, 2) address stigma 3) are tailored to the users’ linguistic and cultural backgrounds, and 4) involve long-term, multi-component programmes co-created with disadvantaged communities.

**Further considerations and implications:**

To improve effectiveness, policies should target disadvantaged groups, address linguistic and cultural barriers, co-create interventions with communities, and combat stigma through tailored approaches and professional training. Sustainable, long-term support mechanisms are needed to maintain health benefits and reduce inequalities in behavioural intervention outcomes. Current evidence offers limited insights into specific intervention components and their differential impacts across diverse groups. Future research should address these gaps to provide more specific guidance for policy and practice.

## Highlights

• Behavioural interventions are often less effective for disadvantaged groups, potentially exacerbating health inequalities.
• Targeted co-created interventions can address cultural, linguistic, and socio-economic access barriers.
• Professional training on stigma reduction and culturally competent care enhance patient trust and intervention effectiveness.
• Long-term, multi-component programs, including peer-led and community-based support, show promise for disadvantaged groups.

## 1 Current policy challenges

Chronic diseases such as Type 2 Diabetes, cardiovascular and respiratory conditions, are among the leading contributors to morbidity and mortality [1,2]. Often, they share common modifiable risk factors, including poor diet, smoking, substance misuse, and physical inactivity [3]. Evidence shows that behavioural or lifestyle interventions - aiming to improve health by encouraging changes in behaviour-can reduce such risk factors at an overall population level [4,5]. However, they are differentially effective across population groups, since people who are more socially and financially secure tend to experience greater benefits [3,6,7].

Interventions addressing behavioural risk factors often adopt an individualistic lens. They emphasise informed choice and personal responsibility while assuming that everyone has similar capacity (material, psychological and social) to adopt healthy behaviours [8,9]. This narrow focus leaves the systemic and cultural factors that affect people's realities and behaviours unaddressed and partially explains the limited effectiveness of behavioural interventions among people in deprived areas and/or some ethnic minorities [9]. For example, research has shown that disadvantaged groups are less likely to access training programmes on healthy diets or physical activity due to the programmes' unsuitable timings, language barriers, culturally irrelevant content, or inappropriate communication tools [10]. As a result, despite their potential to improve population health, behavioural interventions can exacerbate health inequalities when designed without accounting for the cultural and material barriers experienced by disadvantaged groups [8,10].

While addressing these barriers requires action across multiple levels from individual behaviour and community norms through to addressing the social determinants of health [11], healthcare systems can still adopt a structurally informed approach in behavioural interventions design and delivery to ensure that these mitigate instead of perpetuate inequalities in health and healthcare [12]. In this review, we discuss international policy-focused evidence on how healthcare systems can respond to this challenge and improve access, uptake and optimisation of behavioural interventions for disadvantaged groups.

## 2 Approach to collating evidence

The purpose of this evidence brief is to draw lessons from the most relevant and robust policy-focused research exploring how to optimise behavioural interventions for disadvantaged groups, rather than identifying all published studies on a broad policy area. We focused on interventions aiming to improve the health of disadvantaged groups by encouraging behavioural changes related to diet, physical activity, smoking habits, or alcohol consumption and/or with chronic disease self-management like blood-pressure monitoring or medication adherence. We focused on disadvantaged groups as described by PROGRESS Plus categorisation [13].

We collated evidence from 153 studies from the HEEC Living evidence maps which uses EPPI review to map what works to address health inequalities [14] and a MEDLINE search (strategy available as supplementary material). We also drew upon guiding principles set out in a realist [15] and an integrative review [16] of interventions decreasing health inequalities through primary care. Most collated studies were systematic reviews or meta-analyses (n = 138). Studies were appraised for inclusion in the synthesis based on their methodological strength (prioritising systematic reviews and meta-analyses covering ten or more primary studies) and direct relevance to the review question (focusing on studies that evaluated the effectiveness of interventions for disadvantaged groups with measurable health outcomes). Based on this, 58 studies were considered of major importance (i.e, large, methodologically robust SR/meta-analysis that directly evaluates interventions for disadvantaged groups with relevant health outcomes) and most of them are cited in this brief. A full list of the reviewed studies is available in the supplementary material.

Findings were synthesised to identify common characteristics of effective interventions, with a focus on improving outcomes among disadvantaged groups. The studies covered diverse localities, settings, and health outcomes, including diabetes, blood pressure and hypertension, asthma, and chronic obstructive pulmonary disease (COPD), and risk factors including body weight and Body Mass Index (BMI), smoking rates, physical activity, and alcohol consumption. Interventions ranged from group-based education to digital tools and community health worker-led programmes. This evidence brief adds value in three ways. First, it integrates evidence from two complementary sources, a living evidence map and a targeted MEDLINE search prioritising recent study, synthesising evidence across multiple intervention types and disadvantaged populations into a single accessible document for healthcare decision makers and providers. Second, by drawing predominantly on systematic reviews and meta-analyses, it provides a higher-order synthesis distilling findings from a far larger body of primary research than the citation count suggests. Third, it translates evidence into actionable recommendations specifically oriented toward healthcare system design.

## 3 Key evidence to inform policy

Despite the breadth of research on behavioural interventions, there is no single set of specific policy initiatives that guarantee increased uptake and optimisation of behavioural interventions for disadvantaged groups. However, common features in the most effective interventions included being: 1) targeted at specific populations; 2) addressing stigma 3) tailored to users’ linguistic, cultural, and socio-economic backgrounds; and 4) long-term, multi-component and co-created with disadvantaged communities. Below, we organise the most important findings across these three areas.

### 3.1 Targeted approach

A systematic review on chronic disease self-management support interventions showed that individuals from lower socio-economic backgrounds are less likely to participate in such programmes [17]. Analysing 19 studies, the authors found that participation rates improve with the use of targeted recruitment strategies, like referrals. Additionally, interventions that consider users’ social contexts and minimise the added workload for them are more likely to lead to positive health outcomes for disadvantaged groups compared with interventions based on self-efficacy models. Based on these findings, the authors suggested the Cumulative Complexity model as an effective framework for clinical practice because it balances patient burden and capacity [18].

Evidence on smoking cessation interventions in the UK further shows the importance of targeted approaches. A systematic review and equity analysis [19] revealed that General Practice (GP) brief interventions can effectively identify and support disadvantaged smokers, with a positive impact on smoking cessation inequalities. Additionally, mobile drop-in ‘stop smoking services’ (SSS) have shown success in engaging first-time users and smokers from routine or manual occupations, as evidenced by a programme in Nottingham City [20]. A review of studies across Europe highlighted that the targeted approach used in the UK compensates for lower quit rates and has an equitable overall impact on smoking prevalence [21].

Targeted recruitment begins with identifying groups at higher risk of health inequalities. An integrative review emphasised the importance of using sensitive, localised approaches to identify such groups and reduce inequalities in primary care [16]. A scoping review of nine studies on physical activity (PA) recruitment strategies showed that knowing the audience was a consistent factor in successful recruitment among populations experiencing barriers, such as culturally diverse groups, older adults, or people with lower socio-economic status [22]. Having a good understanding of the circumstances and needs of the targeted groups allows for better tailoring of the interventions. For example, evidence has shown that health promotion programmes for unemployed individuals work better when participation is voluntary [23], and that smoking cessation programmes for pregnant smokers work better when they are designed with an ‘opt-out’ referral system [24]. Similarly, findings from UK studies on the health checks conducted in the National Health Service (NHS) suggest that outreach services in familiar venues and clear communication about the checks' purpose can encourage attendance and proactive behaviour changes [25,26]. It should be noted that much of this evidence is drawn from studies of people with low socio-economic status or from ethnic minority groups but does not offer more specific insights into the characteristics of those groups.

### 3.2 Addressing stigma

Stigma is a significant barrier for disadvantaged groups engaging with healthcare services, particularly in interventions focused on behaviour change and risk factors like smoking and obesity [8,27,28]. Popular narratives often attribute these behaviours to individual choices made by people who decide independently based on information they have [8]. Consequently, behavioural interventions often prioritise ‘educating’ patients expecting that this will result in individuals making the ‘right’ decisions for their health [9,15]. When such efforts fail, then it is individuals who carry the blame, compounding stigma [28].

Smoking and weight stigma are particularly prevalent in society, including in healthcare services, fueled by stereotypes that portray smokers and people with obesity as weak or irresponsible [8,27,28]. Such stereotypes intersect with socio-economic status, gender, and ethnicity, disproportionately affecting women from low socio-economic or ethnic and racial minority backgrounds [24,[29], [30], [31]]. A review of 23 qualitative studies showed that pregnant women often hide smoking from health professionals to avoid judgment, viewing their care models as paternalistic and reliant on impersonal material like brochures [29]. Additionally, evidence from the UK suggests that pregnant smokers engage more with interventions when these employ personalised outreach and more inclusive techniques, like phone applications, that protect pregnant smokers from feeling judged [24]. Similarly, a meta-synthesis of 46 studies on weight management during pregnancy revealed that interventions including sensitive, evidence-based counselling, and affirmative communication improved intervention uptake and effectiveness [30]. This evidence suggests that empowering healthcare professionals with training on appropriate language use, evidence-based support, and available referral pathways is critical to delivering behavioural interventions which empower instead of stigmatising recipients.

### 3.3 Tailored programmes to users’ linguistic and cultural backgrounds

Aligning health interventions with users’ linguistic, cultural, and socio-economic backgrounds can improve uptake and outcomes among disadvantaged groups. A systematic review and meta-analysis of 31 studies examining diabetes care interventions for African Americans showed that culturally adapted diabetes self-management education reduced Haemoglobin A1c (HbA1c) levels by 0.8% [32]. Similar findings emerged in a meta-analysis of 12 studies involving 1495 participants which demonstrated that culturally tailored diabetes education benefited ethnic minority groups more than standard care, with the most substantial effects observed at six months (−0.41 HbA1c) [33].

Additional evidence shows that culturally appropriate interventions positively impact obesity, cholesterol, and HbA1c levels over short and medium terms among culturally and linguistically diverse (CALD) groups [34]. Cultural tailoring often involves adapting materials to the local dialect and cultural nuances, and community members delivering the education. A review of 74 studies on community-based programmes for black populations [35] identified several effective tailoring strategies, including:

• **Peripheral strategies**, e.g., marketing intervention programmes as culturally specific.
• **Evidential strategies**, e.g., incorporating resources tailored to specific groups like race-specific foot care advice for people with dark skin.
• **Constituent-involving strategies**, e.g., engaging lay individuals, celebrities, or ethnicity-concordant organisations in information sharing or campaigning.
• **Socio-cultural strategies,** e.g., using cultural elements like recipes, dance classes, and family involvement.

Further, a systematic review of diabetes interventions for black populations showed that tailoring across four domains [36] (i.e., location, facilitators, messaging, and language) led to better outcomes, with interventions incorporating all four domains having the greatest impact on HbA1c levels, weight, and diabetes knowledge compared to those addressing only one domain.

Cultural tailoring is equally relevant to digital health interventions. A realist review of digital interventions for cardiovascular disease among South Asian and Black minority ethnic groups showed that acceptability and relatability differed across ethnicities [37]. For example, South Asian participants valued family-centred learning, while African Americans prioritised community-based information sharing. What was commonly appreciated however was digital health programmes that incorporated education modules in simple language.

### 3.4 Long-term, multifaceted programmes co-created with disadvantaged communities

Behavioural interventions are complex, involving multiple components (e.g., outreach activities, counselling), formats (e.g., face-to-face, online), and professionals (e.g., healthcare providers, non-medical staff) [19,38,39]. However, evidence on the most effective components for improving outcomes among disadvantaged groups remains limited due to the diversity of target populations, the interplay of intervention elements, and contextual factors. Additionally, most studies compare complex interventions to usual care rather than evaluating specific components or approaches.

Effective interventions often incorporate staff training to provide supportive care for chronic disease management [40,41]. Successful weight management programmes often combine environmental improvements (e.g., adjustments at home, access to recreational activities), acceptance-based strategies (e.g., a body-positive approach: showing acceptance and appreciation of all body types) [42], and practical activities (e.g., walking) alongside some nutritional advice [43]. Combined diet and physical activity programmes effectively reduce diabetes risk and cardiometabolic factors, particularly when culturally tailored for ethnic minorities or adapted for unemployed individuals [41,44]. For smoking cessation, approaches combining behavioural counselling, pharmacological support, and follow-ups (in-person or phone) are most effective especially for ethnic minority and migrant populations [45,46].

Community involvement in intervention design and delivery is also critical. Evidence highlights that co-creation with community members is almost a pre-condition for the effective tailoring of interventions in terms of language and cultural references. [35,47] Further, community health workers (CHWs) and peer supporters, often trusted within underserved communities, have shown modest effectiveness in managing chronic conditions like diabetes [[48], [49], [50]]. For example, meta-analyses found that CHW-led or peer-supported interventions resulted in small but significant reductions in HbA1c levels (e.g., −0.18% to −0.5%) [51]. Combining professional expertise with community-led efforts can improve outcomes, as demonstrated in studies where team-based approaches achieved greater HbA1c reductions than individual assignments [39]. The evidence on community health workers and peer supporters is particularly well-developed for Latino and South Asian populations. Its applicability to other disadvantaged groups or conditions should be interpreted cautiously.

Finally, duration and follow-up are crucial for sustaining potential health benefits. Most of the reviewed studies focused on short-term (≤6 months) or medium-term (6–12 months) outcomes but highlighted that longer-term approaches are needed to sustain positive changes. For example, a systematic review of diabetes self-management education among Latinx populations found the greatest HbA1c reductions occurred within six months, as behaviour change effects often diminished without ongoing support later on [39]. Longer duration training programmes with high contact intensity also showed better results, particularly in interventions for unemployed people [23] and for chronic disease self-management interventions among socio-economically disadvantaged groups [17].

## 4 Further considerations and implications

### 4.1 Limitations of current evidence

Most reviews compared complex interventions to usual care rather than evaluating the impact of specific intervention components and therefore identifying the impact of specific components is difficult. This limited our capacity to compare the effectiveness of different behavioural approaches. Additionally, we found limited evidence on the differential impacts of interventions across diverse population subgroups. Also, it should be noted that the evidence on cultural tailoring draws predominantly on diabetes-related research, followed by evidence on cardiovascular disease and obesity. Less evidence was identified for conditions such as COPD and asthma and for the effectiveness of cultural tailoring for digital interventions. Future research is needed to address these gaps and inform specific policy solutions. A broader limitation is that our evidence review was intentionally bounded to healthcare-based interventions which meant that policy addressing the social determinants of health and structural drivers of health inequalities was outside our scope, though it remains a critical area for future research. Finally, participatory research methodologies, in which affected communities shape research also warrant greater attention.

### 4.2 Recommendations for policy and practice

The reviewed evidence can be translated into the following specific actionable recommendations for healthcare professionals and decision makers, as well as local authorities:

1. **Target efforts to underserved groups:** use a locally sensitive approach, including population needs assessments and data linkage, to identify groups with unmet needs and tailor behavioural interventions to their socio-economic, cultural and linguistic needs. Design recruitment strategies, such as opt-out referrals and outreach in community venues, that actively lower participation barriers for disadvantaged groups.
2. **Co-create interventions:** Collaborate with target communities to ensure interventions are culturally relevant and responsive to local needs. Engage community health workers, peer supporters, and culturally concordant organisations as partners, and test cultural, linguistic, and format adaptations before implementation.
3. **Address stigma:** Embed training in affirming, non-judgmental communication and evidence-based behaviour change support into the continuing professional development of all healthcare staff delivering or referring to behavioural interventions. Create structured opportunities for healthcare teams to reflect on and address implicit biases.
4. **Ensure sustainability:** Commission programmes with a minimum duration sufficient to sustain behaviour change with long-term community-based support, follow-up contacts and refresher sessions, to maintain health improvements.

## 5 Conclusion

To reduce health inequalities, behavioural interventions must evolve beyond ‘one-size-fits-all' approaches. By targeting efforts in improving outcomes among disadvantaged groups, incorporating inclusive, empowering design and communication principles, using long-term planning, and community engagement, behavioural programmes can better meet the needs of disadvantaged populations.

## Ethics statement and consent to participate

Not applicable, as this study is based on a literature review and does not involve human participants.

## Availability of data and materials

The data supporting this article's conclusions are available in the referenced studies.

## Authors' contributions

AG and JF conceptualized the study. AG, SH, HP, AGh, and IK conducted the literature review and data extraction. AG, SH, HP, ADL, PT, AV, SE, JB, LJ and DL contributed to the interpretation of findings. AG led the manuscript development. All authors contributed to and approved the final manuscript.

## Disclosure statement

This report is independent research supported by the National Institute for Health and Care Research ARC North Thames and NHS England. The views expressed in this publication are those of the author(s) and not necessarily those of the National Institute for Health and Care Research, NHS England, or the Department of Health and Social Care.

## Funding

This study was commissioned by NHS England.

## Declaration of competing interest

The author is an Editorial Board Member/Editor-in-Chief/Associate Editor/Guest Editor for this journal and was not involved in the editorial review or the decision to publish this article.

The authors declare the following financial interests/personal relationships which may be considered as potential competing interests:This report is independent research supported by the National Institute for Health and Care Research ARC North Thames and NHS England. The views expressed in this publication are those of the author(s) and not necessarily those of the National Institute for Health and Care Research, NHS England, or the Department of Health and Social Care.
