# Supplementary material for: Improving behavioural interventions in healthcare to address the needs of disadvantaged groups: A policy-focused evidence brief
Source: Public Health Pract (Oxf). 2026 Mar 26;11:100780. doi: 10.1016/j.puhip.2026.100780 (PMC13247692; doi:10.1016/j.puhip.2026.100780)
Supplement: Multimedia component 1 [file mmc1.docx]

[SR and revised prady and lifestyle and outcome and programme - but not surgery or LMIC]

<https://ovidsp.ovid.com/athens/ovidweb.cgi?T=JS&NEWS=N&PAGE=main&SHAREDSEARCHID=70qwjrYdFy16KOMGD61bSmunuo06UJB9nCd0tspLoua3GalbDipEuDTV3PcMMGgYt>

Ovid MEDLINE(R) and Epub Ahead of Print, In-Process, In-Data-Review & Other Non-Indexed Citations, Daily and Versions <1946 to July 31, 2024>

1 Meta-Analysis as Topic/ or meta analy$.tw. or metaanaly$.tw. or Meta-Analysis/ or (systematic adj (review$1 or overview$1)).tw. or exp Review Literature as Topic/ or cochrane.ab. or embase.ab. or (psychlit or psyclit).ab. or (psychinfo or psycinfo).ab. or (cinahl or cinhal).ab. or science citation index.ab. or bids.ab. or cancerlit.ab. or reference list$.ab. or bibliograph$.ab. or hand-search$.ab. or relevant journals.ab. or manual search$.ab. or selection criteria.ab. or data extraction.ab. or review.pt. or Review/ [systematic review SR filter] 3712775

2 Residence Characteristics/ or Environment design/ or exp Marital status/ or neighbo?rhood*.mp. or residential environment*.mp. or rural*.mp. or inner?city.mp. or housing instability.mp. or housing insecurity.mp. or housing strain.mp. or housing security.mp. or mortgage problems.mp. or foreclosure.mp. or eviction*.mp. or housing loss.mp. or home repossession*.mp. or home ownership.mp. or (repossess* adj3 hous*).mp. or (repossess* adj3 propert*).mp. or mortgage delinquency.mp. or mortgage arrears.mp. or mortgage debt*.mp. or overcrowding.mp. or (living adj1 (outside or inside or near* or adjacent)).mp. or (household adj2 size).mp. or (marital status or marriage status).mp. or (widow* or cohabit* or divorce* or single parent* or live* alone).mp. 379859

3 Cultural Deprivation/ or Acculturation/ or Culture/ or Cross-Cultural Comparison/ or Cultural Characteristics/ or Cultural Diversity/ or Language/ or "Transients and Migrants"/ or exp "Emigrants and Immigrants"/ or Minority groups/ or Minority health/ or Prejudice/ or Racism/ or Xenophobia/ or Social Discrimination/ or exp Race Relations/ or exp Ethnic Groups/ or exp Continental Population Groups/ or Refugees/ or minorit*.mp. or migration background.mp. or racial.mp. or racism.mp. or ethnology.mp. or race.mp. or ethnic*.mp. or non?English.mp. or language other than.mp. or latino*.mp. or latina*.mp. or hispanic*.mp. or whites.mp. or caucasian*.mp. or non?white.mp. or Torres Strait Islander.mp. or aboriginal.mp. or native american.mp. or inuit.mp. or eskimo.mp. or first nation*.mp. or indigenous.mp. or english as a second language.mp. or foreign language.mp. 878613

4 Occupations/ or Unemployment/ or occupations.mp. or unemployment.mp. 68943

5 exp Gender Identity/ or Women's Health/ or gender differences.mp. or (sex disparit* or sex difference?).mp. or gender identity.mp. or sex role.mp. or wom#n* role?.mp. or m#n* role?.mp. or gender* role?.mp. or servicewomen.mp. or Sex factors/ 409174

6 exp Educational status/ or Education/ or Schooling.mp. or educational status.mp. or (education* adj2 level?).mp. or ((higher or better or worse or less) adj educated).mp. or ((higher or better or worse or less) adj level? of education).mp. 162395

7 Religion/ or religi*.mp. 75874

8 Social determinants of Health/ or Psychosocial Deprivation/ or Sociological Factors/ or Working Poor/ or Hierarchy, Social/ or disparit*.mp. or inequalit*.mp. or inequit*.mp. or equity.mp. or deprivation.mp. or gini.mp. or concentration index.mp. or Socioeconomic Factors/ or Social Welfare/ or exp Social Class/ or exp Poverty/ or Income/ or Social class*.mp. or social determinants.mp. or social status.mp. or social position.mp. or social background.mp. or social circumstance*.mp. or socio-economic.mp. or socioeconomic.mp. or sociodemographic.mp. or socio-demographic.mp. or SES.mp. or disadvantaged.mp. or impoverished.mp. or poverty.mp. or economic level.mp. or assets index.mp. or income*.mp. 899979

9 Social Stigma/ or social capital/ or Social Control, Informal/ or exp Social Support/ or exp Social Environment/ or Trust/ or Social conditions/ or Social isolation/ or Social marginalization/ or Anomie/ or social participation/ or social exclusion.mp. or (social adj (capital or cohes* or organis* or organiz*)).mp. or (community adj3 (cohes* or participa*)).mp. or ((neighbourhood or neighborhood) adj cohes*).mp. or social relationships.mp. or social network*.mp. or collective efficacy.mp. or civil society.mp. or informal social control.mp. or neighbo*rhood disorder.mp. or social disorgani?ation.mp. or anomie.mp. or social support.mp. or social participation.mp. or trust.mp. or emotional support.mp. or psychosocial support.mp. or community capital.mp. or neighbo*rhood cohesion.mp. or social influence.mp. or (soci*context* or soci*-context*).mp. 362479

10 Health Status Disparities/ or Health Services Accessibility/ or Health Equity/ or health*care disparit*.mp. or health care disparit*.mp. or health status disparit*.mp. or health disparit*.mp. or health inequalit*.mp. or health inequit*.mp. or medically underserved.mp. 162289

11 or/2-10 [prady specific terms] 2555573

12 ((Gypsy* or gypsies or gipsy* or gipsies) not (moth or moths)).ti,ab. 2035

13 (Roma or romas or romany or romani or romanis or romanies or romanian).ti,ab. 6068

14 (arli or arlis or ashkali or ashkalis or aurari or auraris or balkan egyptian or balkan egyptians or bashalde or bashaldes or boyash* or churari or churaris or cigano or ciganos or erlide or erlides or gitano or gitanos or gitans or horahane or horahanes or kalderash* or lalleri or lalleris or lingurari or linguraris or lovari or lovaris or ludar or ludars or ludari or ludaris or luri or luris or machvaya or machvayas or manouche or manouches or manush or manushs or manushes or modgar or modgars or modyar or modyars or romanichal or romanichals or romanichel or romanichels or romanis?l or romanis?ls or romungro or romungros or rudari or rudaris or tsigane or tsiganes or ungaritza or ungaritzas or ursari or ursaris or yerlii or yerliis or zl?tari or zl?taris).ti,ab. 70

15 (sinti or sinta or sinte or sintis or sintas or sintes).ti,ab. 35

16 (Ceardannan* or (yenish* or yeniche* or jenische*) or (quinqui* or mercheros*)).ti,ab. 67

17 (fairground* or fair-ground* or funfair* or fun-fair* or showmen* or show-men* or sho?women* or show-women* or showperson* or show-person* or showpeople* or show-people* or show communit* or show travel?er*).ti,ab. 341

18 (circus* or (bargee* or canal boat* or barge* or boat-dwell*) or (pavee* or minceir* or lucht* or luchd* or itinerant*) or (travel?er* and (communit* or family or families or irish or ireland* or eire or wales or welsh or scottish or scotland* or highland* or norwegian* or norway* or newage or new-age or itinerant* or minorit* or ethnic* or halting site* or caravan*)) or (travel?ing adj5 (communit* or family or families or irish or ireland* or eire or wales or welsh or scottish or scotland* or highland* or norwegian* or norway* or newage or new-age or itinerant* or minorit* or ethnic* or site* or caravan*))).ti,ab. 4660

19 or/12-18 [traveller terms] 12913

20 (homeless* or (rough adj sleep*) or vagrant* or (street adj (person* or people* or youth* or child*)) or sofa-surf* or sofa surf* or sofasurf* or unstably housed or housing instability or runaway* or refugee* or asylum* or migrant* or immigrant* or (displaced adj (people* or person*))).ti,ab. or exp homeless persons/ or homeless youth/ or exp refugees/ 91274

21 or/2-10,12-18,20 [prady filter] 2591597

22 exp Exercise/ 259708

23 exp Sports/ 225059

24 exp Diet/ 341788

25 exp Obesity/ 273804

26 exp Weight Loss/ 51774

27 exp Smoking Prevention/ or exp Smoking Cessation/ or exp Smoking/ or exp Smoking Reduction/ 184988

28 exp Alcohol Drinking/ 80181

29 (Exercise or physical* activ* or sport or diet or obesity or weight reduction or weight loss or (smoking adj3 (cessation or reduc* or prevent*)) or alcohol).ti,ab,kw,kf. 1549066

30 or/22-29 [lifestyle] 2044192

31 exp Health Services Accessibility/ 138773

32 exp Patient Satisfaction/ 102008

33 exp Hospitalization/ 304235

34 exp Patient Readmission/ 23985

35 exp "Appointments and Schedules"/ 23585

36 exp Unemployment/ 7936

37 exp Sick Leave/ 6955

38 exp "Global Burden of Disease"/ 2499

39 ((access * adj3 (healthcare or health care)) or patient experience* or admission* or admit* or readmi* or hospitali* or attendance or employ* or unemploy* or lost days or sick leave or "time off").ti,ab,kw,kf. 2438218

40 or/31-37,39 [outcome] 2729708

41 1 and 21 and 30 and 40 [SR and revised prady and lifestyle and outcome] 4945

42 limit 41 to yr="2018 -Current" 2248

43 (program* or intervention* or initiative* or policy or policies or service* or (behavio?r adj3 chang*) or (lifestyle adj3 change*)).ti,ab,kw,kf. 3270415

44 exp Health Promotion/ 87361

45 exp Psychosocial Intervention/ 1196

46 Policy/ 10200

47 or/43-46 [programme terms] 3308290

48 1 and 21 and 30 and 40 and 47 [SR and revised prady and lifestyle and outcome and programme] 3034

49 surgery.ti,ab,kw,kf. 1575084

50 48 not 49 2912

51 afghanistan/ or africa/ or africa, northern/ or africa, central/ or africa, eastern/ or "africa south of the sahara"/ or africa, southern/ or africa, western/ or albania/ or algeria/ or andorra/ or angola/ or "antigua and barbuda"/ or argentina/ or armenia/ or azerbaijan/ or bahamas/ or bahrain/ or bangladesh/ or barbados/ or belize/ or benin/ or bhutan/ or bolivia/ or borneo/ or "bosnia and herzegovina"/ or botswana/ or brazil/ or brunei/ or bulgaria/ or burkina faso/ or burundi/ or cabo verde/ or cambodia/ or cameroon/ or central african republic/ or chad/ or exp china/ or comoros/ or congo/ or cote d'ivoire/ or croatia/ or cuba/ or "democratic republic of the congo"/ or cyprus/ or djibouti/ or dominica/ or dominican republic/ or ecuador/ or egypt/ or el salvador/ or equatorial guinea/ or eritrea/ or eswatini/ or ethiopia/ or fiji/ or gabon/ or gambia/ or "georgia (republic)"/ or ghana/ or grenada/ or guatemala/ or guinea/ or guinea- bissau/ or guyana/ or haiti/ or honduras/ or independent state of samoa/ or exp india/ or indian ocean islands/ or indochina/ or indonesia/ or iran/ or iraq/ or jamaica/ or jordan/ or kazakhstan/ or kenya/ or kosovo/ or kuwait/ or kyrgyzstan/ or laos/ or lebanon/ or liechtenstein/ or lesotho/ or liberia/ or libya/ or madagascar/ or malaysia/ or malawi/ or mali/ or malta/ or mauritania/ or mauritius/ or mekong valley/ or melanesia/ or micronesia/ or monaco/ or mongolia/ or montenegro/ or morocco/ or mozambique/ or myanmar/ or namibia/ or nepal/ or nicaragua/ or niger/ or nigeria/ or oman/ or pakistan/ or palau/ or exp panama/ or papua new guinea/ or paraguay/ or peru/ or philippines/ or qatar/ or "republic of belarus"/ or "republic of north macedonia"/ or romania/ or exp russia/ or rwanda/ or "saint kitts and nevis"/ or saint lucia/ or "saint vincent and the grenadines"/ or "sao tome and principe"/ or saudi arabia/ or serbia/ or sierra leone/ or senegal/ or seychelles/ or singapore/ or somalia/ or south africa/ or south sudan/ or sri lanka/ or sudan/ or suriname/ or syria/ or taiwan/ or tajikistan/ or tanzania/ or thailand/ or timor-leste/ or togo/ or tonga/ or "trinidad and tobago"/ or tunisia/ or turkmenistan/ or uganda/ or ukraine/ or united arab emirates/ or uruguay/ or uzbekistan/ or vanuatu/ or venezuela/ or vietnam/ or west indies/ or yemen/ or zambia/ or zimbabwe/ 1359081

52 "Organisation for Economic Co-Operation and Development"/ 616

53 australasia/ or exp australia/ or austria/ or baltic states/ or belgium/ or exp canada/ or chile/ or colombia/ or costa rica/ or czech republic/ or exp denmark/ or estonia/ or europe/ or finland/ or exp france/ or exp germany/ or greece/ or hungary/ or iceland/ or ireland/ or israel/ or exp italy/ or exp japan/ or korea/ or latvia/ or lithuania/ or luxembourg/ or mexico/ or netherlands/ or new zealand/ or north america/ or exp norway/ or poland/ or portugal/ or exp "republic of korea"/ or "scandinavian and nordic countries"/ or slovakia/ or slovenia/ or spain/ or sweden/ or switzerland/ or turkey/ or exp united kingdom/ or exp united states/ 3576565

54 exp European Union/ 18097

55 Developed Countries/ 21593

56 or/52-55 3592997

57 51 not 56 1267735

58 (afghanistan or albania or algeria or american samoa or angola or "antigua and barbuda" or antigua or barbuda or argentina or armenia or armenian or aruba or azerbaijan or bahrain or bangladesh or barbados or republic of belarus or belarus or byelarus or belorussia or byelorussian or belize or british honduras or benin or dahomey or bhutan or bolivia or "bosnia and herzegovina" or bosnia or herzegovina or botswana or bechuanaland or brazil or brasil or bulgaria or burkina faso or burkina fasso or upper volta or burundi or urundi or cabo verde or cape verde or cambodia or kampuchea or khmer republic or cameroon or cameron or cameroun or central african republic or ubangi shari or chad or chile or china or colombia or comoros or comoro islands or iles comores or mayotte or democratic republic of the congo or democratic republic congo or congo or zaire or costa rica or "cote d'ivoire" or "cote d' ivoire" or cote divoire or cote d ivoire or ivory coast or croatia or cuba or cyprus or czech republic or czechoslovakia or djibouti or french somaliland or dominica or dominican republic or ecuador or egypt or united arab republic or el salvador or equatorial guinea or spanish guinea or eritrea or estonia or eswatini or swaziland or ethiopia or fiji or gabon or gabonese republic or gambia or "georgia (republic)" or georgian or ghana or gold coast or gibraltar or greece or grenada or guam or guatemala or guinea or guinea bissau or guyana or british guiana or haiti or hispaniola or honduras or hungary or india or indonesia or timor or iran or iraq or isle of man or jamaica or jordan or kazakhstan or kazakh or kenya or "democratic people's republic of korea" or republic of korea or north korea or south korea or korea or kosovo or kyrgyzstan or kirghizia or kirgizstan or kyrgyz republic or kirghiz or laos or lao pdr or "lao people's democratic republic" or latvia or lebanon or lebanese republic or lesotho or basutoland or liberia or libya or libyan arab jamahiriya or lithuania or macau or macao or republic of north macedonia or macedonia or madagascar or malagasy republic or malawi or nyasaland or malaysia or malay federation or malaya federation or maldives or indian ocean islands or indian ocean or mali or malta or micronesia or federated states of micronesia or kiribati or marshall islands or nauru or northern mariana islands or palau or tuvalu or mauritania or mauritius or mexico or moldova or moldovian or mongolia or montenegro or morocco or ifni or mozambique or portuguese east africa or myanmar or burma or namibia or nepal or netherlands antilles or nicaragua or niger or nigeria or oman or muscat or pakistan or panama or papua new guinea or new guinea or paraguay or peru or philippines or philipines or phillipines or phillippines or poland or "polish people's republic" or portugal or portuguese republic or puerto rico or romania or russia or russian federation or ussr or soviet union or union of soviet socialist republics or rwanda or ruanda or samoa or pacific islands or polynesia or samoan islands or navigator island or navigator islands or "sao tome and principe" or saudi arabia or senegal or serbia or seychelles or sierra leone or slovakia or slovak republic or slovenia or melanesia or solomon island or solomon islands or norfolk island or norfolk islands or somalia or south africa or south sudan or sri lanka or ceylon or "saint kitts and nevis" or "st. kitts and nevis" or saint lucia or "st. lucia" or "saint vincent and the grenadines" or saint vincent or "st. vincent" or grenadines or sudan or suriname or surinam or dutch guiana or netherlands guiana or syria or syrian arab republic or tajikistan or tadjikistan or tadzhikistan or tadzhik or tanzania or tanganyika or thailand or siam or timor leste or east timor or togo or togolese republic or tonga or "trinidad and tobago" or trinidad or tobago or tunisia or turkey or turkmenistan or turkmen or uganda or ukraine or uruguay or uzbekistan or uzbek or vanuatu or new hebrides or venezuela or vietnam or viet nam or middle east or west bank or gaza or palestine or yemen or yugoslavia or zambia or zimbabwe or northern rhodesia or global south or africa south of the sahara or sub-saharan africa or subsaharan africa or africa, central or central africa or africa, northern or north africa or northern africa or magreb or maghrib or sahara or africa, southern or southern africa or africa, eastern or east africa or eastern africa or africa, western or west africa or western africa or west indies or indian ocean islands or caribbean or central america or latin america or "south and central america" or south america or asia, central or central asia or asia, northern or north asia or northern asia or asia, southeastern or southeastern asia or south eastern asia or southeast asia or south east asia or asia, western or western asia or europe, eastern or east europe or eastern europe or developing country or developing countries or developing nation? or developing population? or developing world or less developed countr* or less developed nation? or less developed population? or less developed world or lesser developed countr* or lesser developed nation? or lesser developed population? or lesser developed world or under developed countr* or under developed nation? or under developed population? or under developed world or underdeveloped countr* or underdeveloped nation? or underdeveloped population? or underdeveloped world or middle income countr* or middle income nation? or middle income population? or low income countr* or low income nation? or low income population? or lower income countr* or lower income nation? or lower income population? or underserved countr* or underserved nation? or underserved population? or underserved world or under served countr* or under served nation? or under served population? or under served world or deprived countr* or deprived nation? or deprived population? or deprived world or poor countr* or poor nation? or poor population? or poor world or poorer countr* or poorer nation? or poorer population? or poorer world or developing econom* or less developed econom* or lesser developed econom* or under developed econom* or underdeveloped econom* or middle income econom* or low income econom* or lower income econom* or low gdp or low gnp or low gross domestic or low gross national or lower gdp or lower gnp or lower gross domestic or lower gross national or lmic or lmics or third world or lami countr* or transitional countr* or emerging econom* or emerging nation?).ti,ab,sh,kf. 2558465

59 57 or 58 [LMIC] 2659997

60 50 not 59 [SR and revised prady and lifestyle and outcome and programme - but not surgery or LMIC] 2326

61 limit 60 to yr="2018-current" 1092
